# Testing Three Species Distribution Modelling Strategies to Define Fish Assemblage Reference Conditions for Stream Bioassessment and Related Applications

**DOI:** 10.1371/journal.pone.0146728

**Published:** 2016-01-12

**Authors:** Peter M. Rose, Mark J. Kennard, David B. Moffatt, Fran Sheldon, Gavin L. Butler

**Affiliations:** 1 Australian Rivers Institute, Griffith University, Nathan, Australia; 2 Department of Science, Information Technology and Innovation, Ecosciences Precinct, Brisbane, Australia; 3 NSW Department of Primary Industries, Grafton Fisheries Centre, Grafton, Australia; Ecole normale superieure de Lyon, FRANCE

## Abstract

Species distribution models are widely used for stream bioassessment, estimating changes in habitat suitability and identifying conservation priorities. We tested the accuracy of three modelling strategies (single species ensemble, multi-species response and community classification models) to predict fish assemblages at reference stream segments in coastal subtropical Australia. We aimed to evaluate each modelling strategy for consistency of predictor variable selection; determine which strategy is most suitable for stream bioassessment using fish indicators; and appraise which strategies best match other stream management applications. Five models, one single species ensemble, two multi-species response and two community classification models, were calibrated using fish species presence-absence data from 103 reference sites. Models were evaluated for generality and transferability through space and time using four external reference site datasets. Elevation and catchment slope were consistently identified as key correlates of fish assemblage composition among models. The community classification models had high omission error rates and contributed fewer taxa to the ‘expected’ component of the taxonomic completeness (O/E_50_) index than the other strategies. This potentially decreases the model sensitivity for site impact assessment. The ensemble model accurately and precisely modelled O/E_50_ for the training data, but produced biased predictions for the external datasets. The multi-species response models afforded relatively high accuracy and precision coupled with low bias across external datasets and had lower taxa omission rates than the community classification models. They inherently included rare, but predictable species while excluding species that were poorly modelled among all strategies. We suggest that the multi-species response modelling strategy is most suited to bioassessment using freshwater fish assemblages in our study area. At the species level, the ensemble model exhibited high sensitivity without reductions in specificity, relative to the other models. We suggest that this strategy is well suited to other non-bioassessment stream management applications, e.g., identifying priority areas for species conservation.

## Introduction

Species distribution models (SDMs) are widely used for stream bioassessment, estimating changes in habitat suitability and identifying conservation priorities, and the accuracy of these models is critical for guiding effective stream management decisions. SDMs relate known species occurrences to landscape, climate and habitat variables to predict species occurrence probabilities across the landscape or river network [1, 2]. SDMs are used for several important stream management applications, most commonly the prediction of reference assemblages for bioassessment [3, 4, 5], but also for quantifying species-environment relationships to inform management [6, 7]; assessing assemblage changes under different land use patterns [8]; predicting responses to future climate or restoration scenarios [7,9]; mapping aquatic biodiversity and identifying species conservation priorities [10, 11, 12]; predicting the invasion potential of alien or translocated species [13, 14, 15]; and identifying remnant populations and uncovering species range extensions or gaps [16].

Stream bioassessment using SDMs usually relies on a reference condition approach, whereby the taxa observed at a test site are compared to modelled taxa predicted to occur at an environmentally similar site, derived from a regional pool of minimally impacted sites [3,4,5]. Both the quality and representativeness of selected reference sites and the modelling strategy applied can affect the success of bioassessment using the reference condition approach [5, 16]. The present study focusses on the effectiveness of different modelling strategies for stream bioassessment and complementary management applications.

Three modelling strategies are widely used for predicting species distributions, each reflecting different theories about how assemblages are structured, and each having strengths and weaknesses in their implementation. The River Invertebrate Prediction and Classification System (RIVPACS) community modelling approach is the most widely used strategy for stream bioassessment [3, 17]. RIVPACS is an ‘assemble then predict’ strategy [18] and relies on a classification step which is most often undertaken using discriminant function analysis (DFA). The need to classify biotic data into discrete assemblages has been perceived by some as an artificial construct, because these rarely occur in nature [19, 20]. In the RIVPACS approach, taxa predictions at new sites are not derived from a single predefined assemblage; instead, the prediction system uses weighted average smoothing across groups to predict the biotic assemblage at sites intermediate in character to those on which the model is derived [21]. This enables RIVPACS models to predict taxa across a continuum of ‘stream types’. Perceived drawbacks include its relatively strict statistical assumptions and multiple steps and decisions involved with the classification procedure [22]; reduced ability to model complex, non-linear ecological data; and that individualistic species responses to key environmental gradients are not able to be directly modelled [20]. A recent advance to overcome some statistical limitations of DFA in RIVPACS models is the use of more flexible machine learning (ML) algorithms for the classification step (e.g. random forest–see [23]). Recently however, other less restrictive strategies have been explored for predicting aquatic taxa, falling broadly into either the category of ‘predict then assemble’, also termed single species models (e.g. [17, 21]), or ‘predict and assemble together’, also termed multi-species response models [16, 20, 24, 25].

The ‘predict then assemble’ strategy involves individually modelling each species and assembling them to synthesize a community at a site. The basis for single species SDMs is grounded in ecological niche theory [26] and usually relies solely on autecological correlative relationships. This strategy allows selection of the best suite of predictor variables relevant to each species [27], rather than those that provide the best average response over an entire assemblage. Alongside traditional logistic regression, a large number of ML algorithms have been used to model individual species distributions and these are advantageous through their flexibility (e.g. ability to fit non-linear functions; automatically fit interactions) and ability to model complex data responses. Furthermore, combining predictions of several of the most accurate models across different algorithms (ensemble models–[28, 29]) may result in more accurate predictions when applied to external validation datasets [30, 31]. The use of ensemble models in aquatic studies is recent, and while this approach has been used to model freshwater taxa under climate change scenarios [32], conservation and stream bioassessment applications are rare (but see [33, 34]). A potential drawback of the ‘predict then assemble’ strategy is that co-occurrence data is not explicitly used in the modelling process and therefore the potential influence of biotic interactions (e.g. via competitors, predators, prey, parasites) is not inherently included [35]. A practical limitation is that it can be laborious to separately model each species, particularly for taxa rich groups (e.g. macroinvertebrates, phytoplankton). Although with advances in statistical software, this is becoming less of a constraint.

The third strategy, ‘predict and assemble together’ reflects the theory that communities assemble with elements of both discrete community and individualistic species responses to environmental gradients [20]. Multivariate adaptive regression splines (MARS) and multiresponse artificial neural networks (MANN) are two methods that reflect this view and both predict an entire assemblage in a single analysis. Some researchers have reported that multi-species response strategies more accurately model rare species because they can ‘borrow’ information from the species-environment relationships of more widespread species [25], while others have found no increase in accuracy or transferability [36, 37]. By constraining selection of variables to those that have a community signal, multi-species response models potentially produce more realistic predictor response curves than their single species ML model counterparts, which often have pronounced discontinuities [36].

SDMs are commonly used to identify important environmental determinants of species distributions by assessing the relative importance of predictor variables and examining the species response curves in partial plots of selected predictor variables [38]. However, apparent species-environment relationships may differ depending on the modelling method used [39]. By assessing differences in variable importance among competing modelling strategies, variables likely to be truly important can be distinguished from those selected due to model idiosyncrasy. Furthermore, because model applications that assess species range changes require spatially continuous data, it is useful to assess the importance of field derived habitat variables, and whether these markedly improve model accuracy compared with GIS-based variables alone [40].

The generality (or transferability) of species-environment relationships within an assessment area is also important for bioassessment. In practice, these relationships might not transfer to different spatial or temporal settings either due to model over-fitting (e.g. excessive parameters with weak correlation to the response variable or fitting the training data to a narrow set of environmental conditions) or model under-fitting, that is, not including key environmental variables in the model [6, 37]. In bioassessment, predictions at new sites need to be accurate so that index results reflect anthropogenic disturbance rather than model error. Furthermore, it is important to quantify the stability of predictions through time to assess whether comparable site assessments can be made among seasons and years (e.g. [5]). Finally, different sampling protocols are used in different jurisdictions, and it is instructive to see whether model predictions are transferable to samples collected using different methods, and thus more widely applicable. Because different modelling strategies may be prone to over- or under-fitting training data, thorough external model validation in different spatial and temporal contexts is required.

Studies comparing the performance of different SDM algorithms are now fairly common (e.g. [1, 41]). However, very few investigations have focussed on the predictive success of the three broadly different modelling strategies previously identified specifically for stream bioassessment (but see [20]). Moreover, there is a paucity of studies that explore the implications of employing these strategies and their method of evaluation to the range of potentially useful applications for stream management [16]. Different evaluation measures highlight the advantages and drawbacks of modelling strategies for different intended applications (2, 16]. Ideally, SDMs have low omission and commission error rates (i.e. high sensitivity and specificity respectively); however, these errors are usually a trade-off. Depending on the intended model use, and costs associated with making incorrect decisions, preferences will be either to balance these errors, or favour one or the other [2]. For example, a model that balances omission and commission errors may be well suited to stream bioassessment, while an alternative model that exhibits high sensitivity (i.e. correctly predicted presence) with some minimum criteria for specificity (correctly predicted absences) may be more useful for identifying suitable areas for re-stocking a species. This is because the model would highlight areas where a species could be (i.e. maximising potentially suitable habitat), rather than where it is presently observed [2]. The emergence of broad scale stream bioassessment programs has resulted in large collections of aquatic species distribution data that may have applications beyond the purpose for which they were originally intended. Comprehensive evaluation of modelling strategies has potential to shift bioassessment programs beyond ‘first-cut’ river health assessments by improving the sensitivity of biotic indices to detecting anthropogenic impacts and by making undervalued monitoring data more widely applicable.

This current study uses fish assemblage data collected at least disturbed reference sites for the Ecosystem Health Monitoring Program (EHMP) (see [42]), to evaluate the accuracy and potential utility of three different modelling strategies (single species, multi-species response, and community classification) for stream bioassessment and other model applications for stream management. Specifically, it aims to: (1) explore species-environment relationships and evaluate consistency of predictor selection among strategies; (2) assess whether the addition of field derived variables to GIS-based variables significantly improves model accuracy; (3) determine which strategy is best suited to stream bioassessment programs using fish indicators and a reference condition approach by externally evaluating model performance in space and time; and (4) appraise which strategies may be suited to other applications that can be used to complement bioassessment programs (e.g. conservation reserve design, assessing species range changes under climate scenarios, identification of suitable re-stocking sites).

## Materials and Methods

### Study area

**Fig 1:**
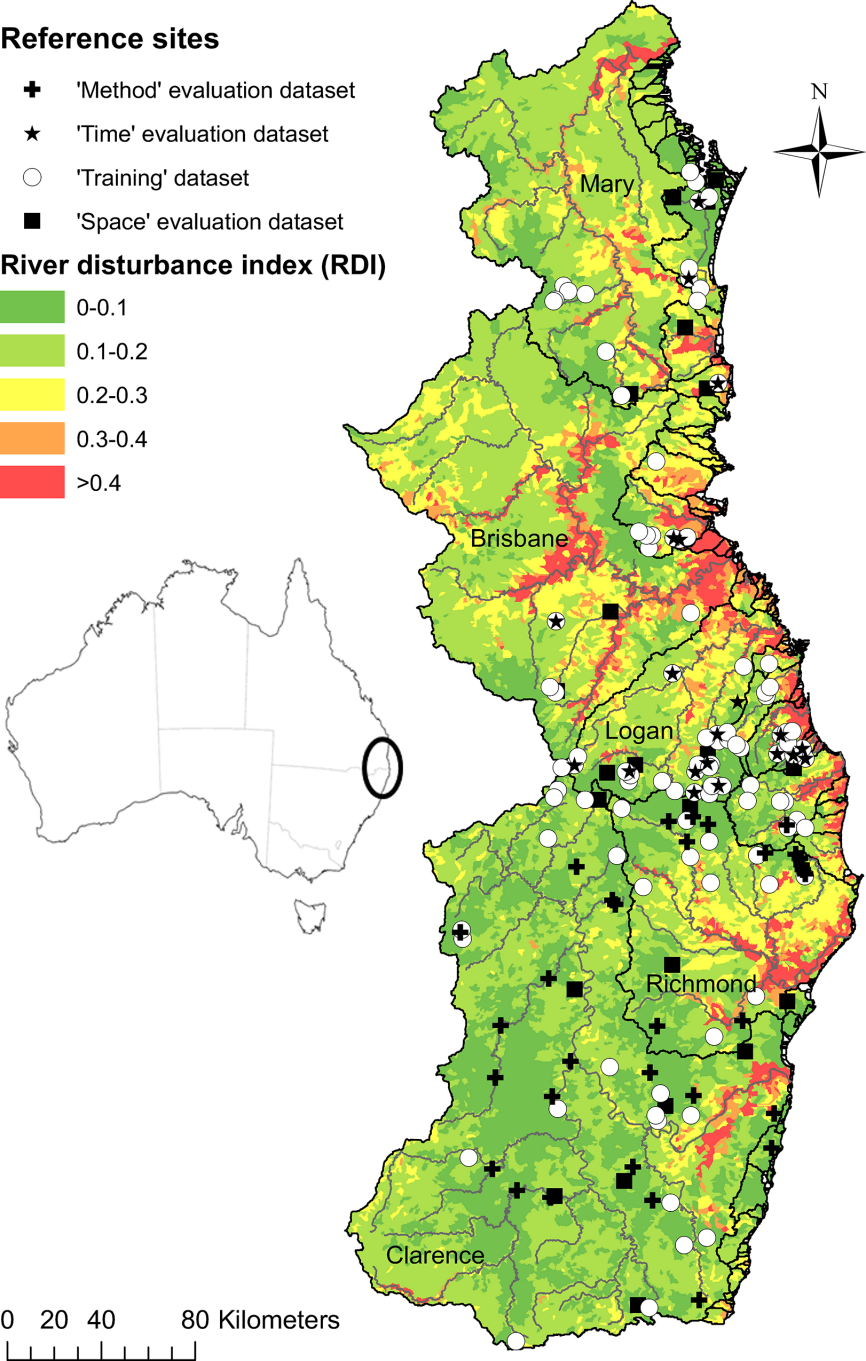
Reference site locations for each dataset used, and major river systems in the study area. The river disturbance index (RDI–see [47] for details of its derivation) provides context for the ‘least disturbed’ reference sites; low RDI values indicate low levels of human pressures in the upstream catchment. Note that the season dataset is represented by all ‘training’ sites in the SEQ section of the study area.

The study area includes streams in sub-tropical eastern Australia extending from the Mary River Basin in south eastern Queensland (SEQ), to the Clarence River Basin in north eastern New South Wales (NEN), Australia, and spans an area of approximately 64,000 km^2^ (Fig 1). It occurs within the eastern biogeographic province [43], and represents a transitional zone for tropical and temperate fish species [44]. The climate ranges from cool-temperate near the Great Dividing Range on the western margin to sub-tropical along the eastern coastal margin. Rainfall and stream flows are generally highest during summer and autumn, although many of the streams exhibit highly variable flow, both seasonally and inter-annually [45]. The area contains several vegetation types including: subtropical; warm and temperate rainforest; wet and dry sclerophyll forest; tableland and dry valley woodlands; and coastal ‘wallum’ (Banksia spp.) heaths [46]. Key land uses in the region include cattle grazing and cropping, large tracts of urban and industrial development, managed and plantation forests and a range of intensive plant and animal industries. Consequently, many streams in the study area have degraded water quality, in-stream habitat and riparian condition. As indicted by spatial patterns in River Disturbance Index scores ([47]; Fig 1), the central and northern section of the study area contains large urbanised areas and has a greater number of regulated stream sections and anthropogenic barriers to fish passage than does the southern section (see [48] for more information on the environmental characteristics of the study area).

### Site selection, sampling methods and datasets

Fish assemblages were sampled at 128 least disturbed reference sites (Fig 1; see [48] for details) in the post wet season (autumn/winter) of 2013 using a standardised single pass backpack electrofishing protocol ([49]; mean stream length of 98m), which yields reliable estimates of species presence-absence at each site. Fish sampling was carried out under Animal Ethics permits (Department of Agriculture and Fisheries Permit SA 2012/11/393; and Griffith University Animal Ethics Permit ENV/04/12/AEC). The study area received average rainfall during the autumn/winter sampling period, following a wetter than average summer. The study area had a reasonable spatial coverage of sampling sites except for the Brisbane River Basin upstream of Wivenhoe Dam, owing to the lack of reference quality sites in this area (Fig 1). Data from 103 sites (80% random subset) were used to train the models (hereafter termed ‘training’ dataset–see Fig 1) and data from the remaining 25 sites (20%) were used as external model evaluation (‘space’ dataset–Fig 1).

Three additional datasets were sourced to externally evaluate model generality through space and time. The first dataset consisted of fish catch from 79 of the 128 previously described SEQ sites, sampled in the pre-wet season (spring/summer) of 2012 (‘season’ dataset). Use of that data enabled assessment of whether seasonality markedly affected predictions, and from a bioassessment perspective, whether a seasonal sampling window is required for data collection at test sites. The second dataset contained 23 sites from SEQ corresponding to those from the training dataset, sampled biannually between 2003 and 2011 (‘time’ dataset–Fig 1) (n = 331). Note that not every site was sampled each season owing to sampling constraints (e.g. depth too great to sample effectively). This allowed assessment of the stability of predictions through time and therefore whether valid comparisons can be made at test sites in the future (i.e. does the benchmark markedly fluctuate with antecedent conditions?). The third dataset contained 33 sites from NEN drawn from the New South Wales Department of Primary Industries freshwater fish database [50] that had been sampled with a single pass electrofishing (backpack or boat) and bait trapping method (‘Method’ dataset–Fig 1). This enabled assessment of whether the model predictions are transferable to samples collected with a different fish sampling protocol, acknowledging that these samples were also collected over a number of seasons and years. The first four datasets (training, space, season and time) were all sampled using a consistent standardised single pass electrofishing protocol [49].

### Fish data

Fish catch data were converted to species presence-absence, and alien and estuarine vagrant species were removed. Species occurring at only one site in the training dataset were also removed, as these could not be modelled by all of the methods. The final training dataset consisted of 25 native species with prevalence among sites ranging from 3% (*Ophisternon sp*.) to 95% (*Anguilla reinhardtii*), with a mean (± SD) fish species richness of 6.5 (±2.8) per site (S1 Table).

### Predictor variables

Twenty-three ecologically relevant predictor variables representing four different spatial scales were selected from a larger list of candidate variables (see S2 Table) as input to modelling. Candidate variables were chosen on the basis of prior research in the region [5, 6, 51]. Variables were excluded if they had missing values or they were highly correlated (absolute Pearson r>0.8). For highly correlated variable pairs, we decided which variable to omit on the basis of which we believed would have a more direct influence on fish assemblages. Twenty variables were GIS-based, and represented three spatial scales: (1) the stream segment and associated sub-catchment, (2) the downstream flow path and (3) the upstream catchment (see [52] for details). The remaining three site-scale predictors; stream width, depth and flow velocity, were measured at each sampling site using standard EHMP methods [49] to form part of the training, space, and season datasets. With these datasets, fish assemblages were modelled with and without the site-scale environmental variables measured in the field to determine whether inclusion of these variables significantly increased model performance. Eight variables were transformed to meet the assumption of a normal distribution required by some models (e.g. DFA) (see S2 Table).

### Model fitting

#### Single species ensemble model (ENS)

Predictive models were constructed and mapped to the relevant portion of the stream network of Stein et al. [52], which consisted of 10,928 stream segments with Strahler stream order>1. Stream segments were defined as the length of stream between two confluences (mapped at 1:250000 resolution), and were typically 2-3km in length. First order stream segments, which were not considered in this study, had an average upstream catchment area of 3.5km^2^. Five types of commonly used single species models were selected to generate a single ensemble model for each species using the *BIOMOD* package [29] in R (version 2.13.1, Foundation for Statistical Computing: Vienna, Austria). The models used were: generalised linear models (GLM), random forest (RF), boosted regression trees (GBM), artificial neural networks (ANN) and multivariate adaptive regression splines (MARS). Parameter settings used for each model were the default in the *BIOMOD* package. Models were trained using 80% of the ‘training’ dataset and k-fold cross validated (k = 5) using the remaining 20%. This process was repeated 10 times for each modelling algorithm, using a randomised 80/20% allocation of sites each time, resulting in 50 candidate models per species for ensemble modelling. Only cross-validated models were retained for the ensemble model, that is, the full model was not considered. The true skill statistic (TSS) was used as the criterion for model retention. TSS equals the sensitivity + specificity– 1 [53]. Models were retained if the TSS>0.8, except where no candidate models met this criterion, in which case, TSS> 0.7 was used. The retained models were combined into a single ensemble model for each species by calculating the arithmetic mean of prediction probabilities. We chose this method as it has been shown to provide more robust predictions than single models and most other consensus techniques [28].

#### Multi-species response models (MARS and MANN)

Two multi-species response models were generated. These models use an input matrix containing all species, and make predictions in a single analysis. The first was a multivariate adaptive regression splines (MARS) multiresponse model [54] and was fitted using custom R scripts [55] and the *mda* package [56]. First order interactions were allowed, and a penalty value of 2 was selected to avoid over-fitting (this parameter penalises degrees of freedom). Other penalty values (0.5, 1, 1.5 and 2.5) were trialled but these model iterations were less successful, as assessed by the mean cross-validated species area under curve of the receiving operator characteristics plot (AUC), and are not reported on further.

The second multi-species response model generated was a single hidden-layer feed-forward multirepsonse artificial neural network (MANN), model fit using the *nnet* package in R [57]. Initially, the performance of different network structures was evaluated using a range of hidden nodes (0–20) and decay weights to optimise the model and prevent over-fitting [57]. The best structure, selected using the highest mean cross-validated species AUC, contained seven hidden nodes and a decay weight of 0.03. Because neural network predictions can differ for different model iterations owing to the assignment of initial random parameter weights, we generated 10 MANN models, with the best fit (evaluated by mean species AUC) retained as the final model. An ensemble MANN was also trialled by retaining the five best models (evaluated by mean species AUC) and calculating the arithmetic mean of probabilities of occurrence; however, this model did not perform as well as the ‘best’ individual model, and is not reported on further.

#### RIVPACS community-type models (DFA and RF)

Two RIVPACS community-type models were constructed. This procedure uses an initial step that classifies the biota into assemblage groups (see [3] for a detailed description of the general modelling procedure). Firstly, Sørensen’s dissimilarity was calculated from the species by site matrix, and sites were clustered using the flexible unweighted pair group method with arithmetic mean (β = -0.6). Six assemblage groups were identified visually from the resultant cluster (see S1 Fig). Secondly, two classifiers were used to predict the group membership of sites, one using a traditional approach (DFA classifier selected using stepwise backwards elimination with Akaike’s information criterion), and the second using a random forest classifier (RF; [58]). Thirdly, occurrence probabilities for each species at each site were calculated by multiplying the site probability of group membership by the species frequency of occurrence for each group, then summing the products across assemblage groups. The underlying group classification was the same for each model. Both models were constructed using custom R scripts [23].

### Relative importance of predictor variables

Methods for assessing predictor variable importance were model specific. Single species ensemble models were assessed by a permutation approach (see [29]). The MARS multi-species response model was assessed using the loss of deviance explained when the variable under consideration was omitted [54]. The MANN model was assessed using the connection-weight approach [20]. The RIVPACS DFA model was assessed using F-to-remove statistic associated with the partial Wilk’s *lambda* for the variable under consideration (e.g. [59]) The RIVPACS RF model was assessed using mean decrease in the Gini index [60]. To enable comparison among models, the average rank importance of retained predictors was used.

### Model evaluation

The match between the predicted and observed assemblage composition was evaluated using two metrics: the taxonomic completeness index (the ratio of observed to expected species) at a cut-off threshold of 0.5 (O/E_50_), and the Bray-Curtis dissimilarity index [61] (Table 1). For O/E_50_, the number of expected species is calculated as the sum of species probabilities >0.5. We selected 0.5 as the cut-off as it is the most commonly used threshold for stream bioassessment. For the Bray-Curtis index, all species were included (i.e. a cut-off threshold of 0).

**Table 1:** Metrics used to evaluate model performance at species and assemblage levels.

Code	Metric	Description
**Species level metrics**		
AUC	Area under curve of the receiving operator characteristics plot	Ranges from 0.5 to 1; Higher values indicate a better fit
Se	Sensitivity	Correct prediction of presence (ranges from 0 to 1; 1 indicates perfect prediction)
Sp	Specificity	Correct prediction of absence (ranges from 0 to 1; 1 indicates perfect prediction)
K	Cohen's Kappa	Generally ranges from 0 to 1; higher values indicate a better fit
CCR	Correct Classification Rate	Proportion of sites correctly classified as either present or absent
**Assemblage level metrics**		
Mean O/E	Mean of O/E index	Indicates model accuracy
Bandwidth	90^th^ percentile minus 10th percentile of O/E index	Estimate of model precision; used to develop 'bands' of impairment
r^2^	Pearson r-squared regression coefficient of O vs E	Model goodness of fit
SD	Standard deviation of O/E index	Estimate of model precision
Slope	Slope of the linear regression of O vs E	Indicative of model bias (e.g. slope <1 indicates underestimation of richness at diverse sites)
Intercept	Y-intercept of linear regression of O vs E	Indicative of model bias
BC	Bray-Curtis dissimilarity index	Dissimilarity between forecast and observed community (i.e. lower is better)
SD BC	Standard deviation of the BC index	Estimate of BC model precision

Model performance was evaluated for each species and dataset using several common SDM evaluation statistics (described in Table 1) generated with the *SDMTools* package in R [62]. For threshold dependant metrics, 0.5 was again used as the occurrence probability cut-off threshold. One-way analysis of variance (ANOVA) and Tukey’s honestly significant difference tests were used to determine if each evaluation metric significantly differed among each of the five models and datasets. To gain insight into the consistency of spatial predictions of each model, we mapped the occurrence predictions of a high prevalence species (*Melanotaenia duboulayi*) and a low prevalence species (*Hypseleotris klunzingeri*).

## Results

### Consistency of predictor variable selection among modelling strategies

Stream elevation was the most influential predictor of fish assemblages, followed by the slope of the upstream catchment, distance to the sea, and mean annual runoff (Table 2). Stream elevation and catchment slope were the only two predictors selected by every model, and the former was ranked as the most important variable by all models except for the RIVPACS DFA. The eight candidate catchment geology variables were all ranked amongst the 10 least important predictors. The models were relatively consistent in predictor variable selection, although the RIVPACS DFA modelling process selected stream temperature in the hottest month and % unconsolidated geology in the upstream catchment as the two most important variables, whereas these variables were not ranked highly for the remaining models.

**Table 2:** Ranking of GIS-based predictor variable importance for predicting fish assemblage composition for each modelling strategy (1 indicates the most important variable).

	Modelling strategy					
Predictor	ENS	DFA	RF	MARS	MANN	Average
Mean segment elevation	1	6	1	1	1	2.0
Catchment average slope	3	3	3	2	6	3.4
Distance to outlet (the sea)	6	7	2	-	3	4.5
Mean annual runoff	2	5	9	-	2	4.5
Catchment shape (elongation ratio)	5	4	7	-	4	5.0
Maximum upstream elevation	4	-	4	-	12	6.7
Stream and sub-catchment average annual rainfall	7	-	8	-	5	6.7
Stream and sub-catchment hottest month mean temperature	12	1	6	-	9	7.0
Average slope of downstream flow path	9	-	10	-	7	8.7
Catchment relief ratio	8	-	5	-	13	8.7
Catchment percentage unconsolidated rocks	16	2	14	-	10	10.5
Catchment percentage igneous rocks	10	-	13	-	14	12.3
Modelled annual terrestrial mean net primary productivity	17	-	12	-	8	12.3
Coefficient of variation of monthly totals of accumulated soil water surplus	11	-	11	-	17	13.0
Stream and valley percentage siliciclastic/undifferentiated sedimentary rocks	13	-	15	-	11	13.0
Stream and valley percentage unconsolidated rocks	15	-	16	-	15	15.3
Catchment percentage metamorphic rocks	14	-	17	-	19	16.7
Stream and valley percentage metamorphic rocks	18	-	19	-	18	18.3
Stream and valley percentage mixed sedimentary and igneous rocks	20	-	20	-	16	18.7
Catchment percentage mixed sedimentary and igneous rocks	19	-	18	-	20	19.0

See S2 Table for predictor variable descriptions. ENS–Single species ensemble model; DFA–RIVPACS community model using a discriminant function classifier; RF–RIVPACS model using a random forest classifier; MARS–Multi-species response multivariate adaptive regression splines model; MANN–Multi-species response artificial neural network model.

### Performance of models with and without field-derived predictors

Predictive accuracy, as assessed by mean species AUC, did not significantly differ between models calibrated using GIS-derived predictor variables and those with the addition of field derived predictors for any of the modelling strategies (p<0.05 for all one-way ANOVAs) (Table 3). Further, none of the 25 species exhibited consistent improvement in AUC with the addition of field derived variables. Given the overall lack of differences, the remainder of the results section relates to the models derived with GIS-derived predictors only.

**Table 3:** AUC averaged among species using (i) all predictor variables and (ii) GIS-based predictors alone, for three datasets.

Model	Variables	Training (n = 103)	Space (n = 25)	Season (n = 79)	Average
ENS	All variables	0.99	0.81	0.86	0.89
	GIS only	0.99	0.82	0.85	0.89
DFA	All variables	0.82	0.77	0.80	0.80
	GIS only	0.81	0.76	0.79	0.79
RF	All variables	0.86	0.81	0.78	0.82
	GIS only	0.86	0.80	0.79	0.82
MARS	All variables	0.85	0.84	0.83	0.84
	GIS only	0.85	0.78	0.83	0.82
MANN	All variables	0.86	0.77	0.74	0.79
	GIS only	0.86	0.76	0.80	0.81

No significant differences (at p<0.05) were detected between (i) and (ii) for any model/dataset combination, assessed by one-way ANOVAs. ENS–Single species ensemble model; DFA–RIVPACS community model using a discriminant function classifier; RF–RIVPACS model using a random forest classifier; MARS–Multi-species response multivariate adaptive regression splines model; MANN–Multi-species response artificial neural network model.

### Accuracy of assemblage composition prediction among modelling strategies

Ensemble models were superior to other modelling strategies across most O/E_50_ evaluation metrics when applied to the training dataset; and generally produced the most precise O/E_50_ estimates across all datasets as indicated by low standard deviation and narrow bandwidths (Table 4). However, mean O/E_50_ and regression slope were consistently <1 when applied to the external datasets implying model bias (i.e. overestimating expected species, particularly for sites with high species richness). The ensemble model also performed poorly for the ‘space’ dataset, driven by a few outlying sites (e.g. O/E_50_ of 0 for a high elevation site in NEN).

**Table 4:** Assemblage level model evaluation metrics typically used to assess model quality for stream bioassessment for each model and dataset.

Dataset	Model	Mean O/E_50_	SD O/E_50_	Band width O/E_50_	r^2^ O/E_50_	Slope O/E_50_	Intercept O/E_50_	Mean BC	SD BC
Training	ENS	1.08	***0*.*13***	***0*.*33***	***0*.*95***	1.11	-0.14	***0*.*20***	***0*.*10***
(n = 103)	DFA	1.03	0.29	0.71	0.61	1.06	-0.09	0.42	0.10
	RF	1.05	0.32	0.61	0.74	1.15	-0.35	0.42	0.10
	MARS	***0*.*98***	0.25	0.57	0.73	0.98	***0*.*01***	0.38	0.10
	MANN	1.06	0.24	0.48	0.87	***1*.*02***	0.17	0.38	0.13
Space	ENS	0.91	0.33	0.72	0.51	0.64	0.85	***0*.*37***	0.15
(n = 25)	DFA	***0*.*99***	0.29	0.56	0.78	***0*.*93***	0.18	0.46	0.13
	RF	1.02	0.30	***0*.*48***	***0*.*83***	1.13	-0.21	0.47	***0*.*12***
	MARS	0.92	***0*.*28***	0.51	0.77	0.90	***0*.*07***	0.42	0.12
	MANN	0.92	0.31	0.62	0.60	0.72	0.52	0.45	0.15
Season	ENS	0.91	***0*.*19***	***0*.*47***	***0*.*78***	0.89	0.06	0.52	***0*.*08***
(n = 79)	DFA	1.01	0.27	0.66	0.60	1.09	-0.30	0.52	0.09
	RF	1.03	0.29	0.62	0.68	1.26	-0.81	0.52	0.09
	MARS	0.97	0.25	0.61	0.69	***0*.*97***	***0*.*05***	***0*.*51***	0.09
	MANN	***1*.*00***	0.26	0.65	0.75	0.96	0.13	0.52	0.10
Time	ENS	0.83	***0*.*24***	***0*.*58***	***0*.*56***	0.71	***0*.*68***	***0*.*37***	0.13
(n = 23; 331 samples)	DFA	1.18	0.34	1.06	0.42	0.78	1.28	0.44	***0*.*11***
	RF	1.16	0.38	0.95	0.43	***0*.*91***	0.85	0.44	0.11
	MARS	***1*.*00***	0.34	0.86	0.40	0.66	1.25	0.42	0.11
	MANN	1.04	0.30	0.71	0.52	0.84	0.78	0.42	0.11
Method	ENS	0.76	***0*.*25***	***0*.*52***	0.50	0.66	0.51	***0*.*41***	0.12
(n = 33)	DFA	0.97	0.30	0.58	0.42	0.78	0.62	0.45	0.11
	RF	***1*.*02***	0.29	0.67	0.49	***0*.*84***	0.55	0.49	***0*.*10***
	MARS	0.93	0.27	0.60	0.43	0.77	0.57	0.43	0.11
	MANN	0.86	0.28	0.57	***0*.*63***	0.71	***0*.*49***	0.47	0.11

Bold and italicised text indicates the ‘best’ metric value for each dataset/model combination. See Table 1 for metric codes and descriptions. ENS–Single species ensemble model; DFA–RIVPACS community model using a discriminant function classifier; RF–RIVPACS model using a random forest classifier; MARS–Multi-species response multivariate adaptive regression splines model; MANN–Multi-species response artificial neural network model.

**Fig 2:**
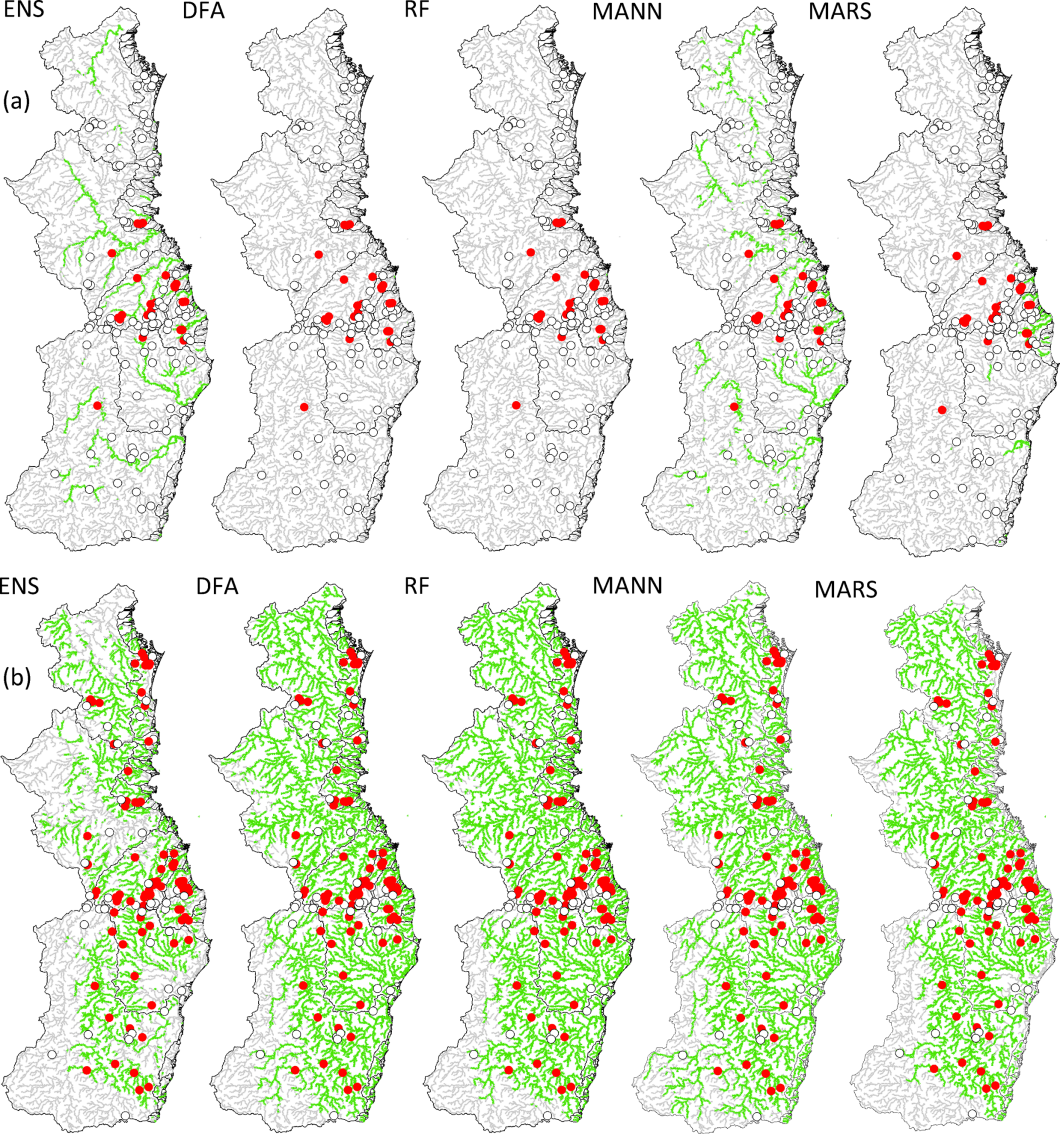
Projected species distributions (at a cut-off threshold of 0.5) for (a) *Hypseleotris klunzingeri* and (b) *Melanotaenia duboulayi*. Green stream segments are predicted presences; grey segments are predicted absences. The circles are sites that were sampled in autumn/winter 2013 (i.e. the training and space datasets; n = 128). Red circles are observed presences, open circles are observed absences. ENS–Single species ensemble model; DFA–RIVPACS community model using a discriminant function classifier; RF–RIVPACS model using a random forest classifier; MANN–Multi-species response artificial neural network model; MARS–Multi-species response multivariate adaptive regression splines model.

The multi-species response and RIVPACS models had similar accuracy (mean O/E_50_ and r^2^) and bias (slope and intercept) across all datasets; however precision (bandwidth and standard deviation) was usually better for the multi-species response models. The RIVPACS models were conservative in including taxa at a 0.5 threshold (e.g. total of nine species were potentially predicted to occur for the training dataset) in comparison to MANN and MARS multirepsonse models (16 and 18 species, respectively) and the ensemble models (all 25 species). For example, the two RIVPACS models did not predict *Hypseleotris klunzingeri*, a low prevalence species (18% in the training dataset), to occur at all (Fig 2A). This species is effectively ignored in the O/E_50_ index, even though it has a highly predictable distribution, as indicated by a mean AUC of 0.9 for the ensemble model (Table 1).

The ensemble model best matched observed to predicted assemblage composition using the BC index relative to other modelling strategies for all datasets except ‘season’ where BC values were almost equivalent among models. The multi-species response models also generally produced lower BC values than the RIVPACS models. The precision of BC had no discernable patterns across datasets or models.

### Accuracy of species predictions among GIS- based modelling strategies

AUC ranged from 0.68 (*Anguilla australis*) to 0.95 (*Hypseleotris compressa* and *Ophisternon* sp.), averaged across datasets and modelling strategies (Table 5). The ensemble model produced good predictions (arbitrarily defined as AUC>0.8) for 20 species compared with MARS (13 species), the two RIVPACS-type models (9 species each) and the MANN model (8 species). On average, the ensemble model most accurately predicted rare species (i.e. those with prevalence <10%); however, differences among strategies were not significant (p>0.05 for one-way ANOVAs). The two RIVPACS-type models least accurately predicted rare species.

**Table 5:** AUC for each species, averaged among datasets. Asterisks denotes rare species (defined as <10% prevalence in the training dataset).

Species	Observed prevalence	ENS	DFA	RF	MARS	MANN	Average
Ambassis agassizii*	9%	0.89	0.75	0.75	0.84	0.85	0.82
*Anguilla australis*	15%	0.78	0.66	0.72	0.63	0.61	0.68
*Anguilla reinhardtii*	95%	0.83	0.84	0.86	0.77	0.79	0.82
*Craterocephalus Marjorie*	13%	0.80	0.81	0.85	0.83	0.78	0.81
Craterocephalus stercusmuscarum*	7%	0.95	0.79	0.78	0.85	0.93	0.86
Galaxias olidus*	6%	0.95	0.82	0.90	0.95	0.96	0.91
*Gobiomorphus australis*	43%	0.97	0.92	0.93	0.93	0.91	0.93
*Gobiomorphus coxii*	29%	0.83	0.71	0.76	0.82	0.77	0.78
*Hypseleotris compressa*	35%	0.94	0.95	0.95	0.95	0.94	0.95
*Hypseleotris galii*	54%	0.84	0.75	0.80	0.79	0.76	0.79
*Hypseleotris klunzingeri*	18%	0.90	0.77	0.71	0.73	0.79	0.78
Leiopotherapon unicolor*	8%	0.73	0.69	0.73	0.69	0.73	0.71
*Melanotaenia duboulayi*	67%	0.78	0.73	0.77	0.74	0.76	0.76
*Mogurnda adspersa*	12%	0.95	0.60	0.64	0.79	0.75	0.74
Mugil cephalus*	9%	0.83	0.85	0.75	0.83	0.74	0.80
*Notesthes robusta*	11%	0.92	0.85	0.77	0.81	0.70	0.81
*Ophisternon sp*.*	3%	0.96	0.93	0.95	0.95	0.95	0.95
*Percalates novemaculeata*	18%	0.77	0.68	0.75	0.69	0.69	0.72
*Philypnodon grandiceps*	26%	0.83	0.74	0.75	0.70	0.79	0.76
*Philypnodon macrostomus*	17%	0.81	0.71	0.79	0.78	0.72	0.76
*Pseudomugil signifier*	14%	0.83	0.69	0.70	0.75	0.71	0.74
*Retropinna semoni*	68%	0.90	0.73	0.84	0.83	0.86	0.83
*Rhadinocentrus ornatus*	15%	0.98	0.93	0.94	0.90	0.97	0.94
*Tandanus tandanus*	55%	0.83	0.73	0.75	0.77	0.73	0.76
Trachystoma petardi*	5%	0.76	0.79	0.72	0.81	0.72	0.76
Number of ‘good’ predictions (AUC>0.8)		19	9	8	13	8	11

ENS–Single species ensemble model; DFA–RIVPACS community model using a discriminant function classifier; RF–RIVPACS model using a random forest classifier; MARS–Multi-species response multivariate adaptive regression splines model; MANN–Multi-species response artificial neural network model.

Model projections varied considerably, including for species with a similar AUC. For example, projections for the relatively common species *Melanotaenia duboulayi* ranged from 40% predicted occupancy of stream segments throughout the stream network for the ensemble models, to 79% for the RF RIVPACS-type model, even though the mean AUC for the two models was almost identical (Fig 2B, Table 5).

Single species ensemble models derived from the training dataset were significantly more accurate (using AUC) than those from other strategies (Table 6). Ensemble models also generalised better across space, season, time and method datasets, although these differences were not significant (Table 6). The ensemble models also had significantly higher kappa, sensitivity, and correct classification rates for the training dataset, as well as the highest values for all evaluation metrics averaged among the datasets. The multi-species response models had intermediate levels of predictive success, while both RIVPACS-type models on average exhibited the lowest success across all of the evaluation metrics. The ensemble models in particular, consistently had the highest sensitivity and consequently lowest omission error rates; however, specificity and correct classification rate did not vary markedly among modelling strategies.

**Table 6:** Mean species level evaluation metrics for each model and dataset.

Dataset (no. sites)	Model	AUC	K	Se	Sp	CCR
Training (n = 103)	ENS	**0.99**^A^	**0.88**^A^	**0.90**^A^	**0.96**	**0.96**^A^
	DFA	0.81^B^	0.16^B^	0.28^B^	0.87	0.86^B^
	RF	0.86^B^	0.17^B^	0.28^B^	0.88	0.87^B^
	MARS	0.85^B^	0.26^B^	0.37^B^	0.87	0.87^B^
	MANN	0.86^B^	0.37^B^	0.43^B^	0.91	0.89^B^
Space (n = 25)	ENS	**0.82**	**0.35**	**0.52**	0.87	**0.88**
	DFA	0.76	0.14	0.29	0.86	0.87
	RF	0.80	0.14	0.29	0.86	0.87
	MARS	0.78	0.18	0.31	**0.88**	0.87
	MANN	0.76	0.20	0.33	0.88	0.87
Season (n = 79)	ENS	**0.85**	**0.49**^A^	**0.62**^A^	0.88	**0.88**
	DFA	0.79	0.14^B^	0.27^B^	0.86	0.86
	RF	0.79	0.14^B^	0.27^B^	0.87	0.86
	MARS	0.83	0.24^B^	0.36^AB^	0.86	0.87
	MANN	0.80	0.25^B^	0.35^AB^	**0.88**	0.87
Time (n = 23; 331 samples)	ENS	**0.81**	**0.36**^A^	**0.62**^A^	0.81	0.83
	DFA	0.73	0.12^B^	0.28^B^	0.85	0.86
	RF	0.74	0.13^B^	0.29^B^	**0.85**	**0.86**
	MARS	0.77	0.13^B^	0.33^AB^	0.82	0.83
	MANN	0.78	0.19^AB^	0.37^AB^	0.84	0.85
Method (n = 33)	ENS	**0.82**	**0.27**	**0.53**	0.84	0.84
	DFA	0.79	0.18	0.33	0.87	0.86
	RF	0.77	0.15	0.31	0.86	0.86
	MARS	0.78	0.20	0.36	**0.87**	**0.86**
	MANN	0.74	0.17	0.37	0.84	0.84
Average	ENS	**0.86**	**0.47**	**0.64**	**0.87**	**0.87**
	DFA	0.78	0.15	0.29	0.86	0.86
	RF	0.79	0.15	0.29	0.86	0.86
	MARS	0.80	0.20	0.35	0.86	0.86
	MANN	0.79	0.24	0.37	0.87	0.87

Significant differences in mean evaluation metrics among models are denoted by different letters (assessed by one-way ANOVAs and Tukey’s HSD tests). Bold values indicate the highest mean evaluation metric for each model/dataset. See Table 2 for metric codes and descriptions. ENS–Single species ensemble model; DFA–RIVPACS community model using a discriminant function classifier; RF–RIVPACS model using a random forest classifier; MARS–Multi-species response multivariate adaptive regression splines model; MANN–Multi-species response artificial neural network model.

## Discussion

### Correlates of fish assemblage composition and consistency of variable selection

Variables related to the site position in the stream continuum (*sensu* Vannote et al. [63]) were strong determinants of fish assemblage composition in our study. These included elevation, catchment slope and mean annual runoff (discharge), and are likely related to stream size and depth, habitat diversity, primary production and carbon sources, water permanency, and the occurrence of important hydraulic habitat types such as pools, runs, and riffles [48]. Consistent with studies in coastal streams both locally [5, 6] and elsewhere [64], distance to the sea was important, as several species require access to the sea to complete their life history (e.g. *Mugil cephalus*, *A*. *reinhardtii*, *Gobiomorphus australis*). Catchment and sub-catchment geology were generally found to be among the least important factors for fish assemblages, which contrasts with several studies elsewhere [24, 41]. Model predictions at headwater sites tended to be the least reliable, and we attribute this to the frequent presence of natural barriers in these stream types (e.g. waterfalls and steep cascades impassable to certain species). Detailed estimates of the size and gradient of such barriers, historical persistence of refugia above these, and frequency of ‘drown-out’ events would likely improve predictions in these areas. This information is currently difficult to acquire at regional scales. However, high resolution topographic data acquired from light detection and ranging (LiDAR) is becoming increasingly available and offers scope for improving model parameterisation for these stream types in the future.

The different modelling strategies identified important variables fairly consistently, with the following exceptions. First, the MARS multi-species response model was exceptionally parsimonious; only identifying two important variables: elevation and upstream catchment slope. Yet this model yielded remarkably good predictive accuracy across all datasets. Second, the DFA RIVPACS model selected air temperature in the hottest month and % unconsolidated geology in the upstream catchment instead of stream elevation, as the most important variables, which was ranked first by all other models. These variables, like elevation, probably serve as a proxy for position in the stream continuum; however, the DFA model generally produced the least accurate predictions. Whether this is due to the predictor selection method (stepwise AIC) or the modelling process (fitting linear functions versus more flexible ML algorithms) is unclear.

### Spatially continuous predictions

Several studies have demonstrated the link between landscape scale predictors, local habitat features and fish assemblage composition in minimally impacted streams [65, 66]. Exploratory studies examining the relative roles of local and landscape variables for structuring fish communities are fairly common (e.g. [67, 68]). However, fewer studies have directly quantified the loss of predictive model accuracy using landscape variables alone, many of which may act as a proxy for local habitat conditions (e.g. [40, 69]). Our study demonstrated that the inclusion of commonly used habitat variables (width, depth and flow velocity) yielded minimal gain in predictive accuracy at a regional scale. However, other work has shown that local habitat variables are important for predicting species occurrence at smaller spatial scales [24], or for predicting other types of species responses such as variation in abundance or biomass (e.g. [6, 70]).

The minimal gain in predictive accuracy using field derived predictor variables has several implications for stream bioassessment programs using fish indicators, which are typically implemented at regional scales. Firstly, the additional collection of field derived variables can be time consuming and expensive, and appears to provide marginal improvements in model accuracy relative to cost [68]. Model accuracy could be improved by instead diverting resources to sampling a greater number of sites or expending a greater sampling effort per site. Secondly, habitat measurements collected at the site are commonly influenced by anthropogenic stressors thereby biasing predictions at test sites [71, 72]. For example, flow velocity and stream depth may be affected by river regulation, water extraction, sedimentation or mobile sand slugs [72]. The use of static, GIS-based landscape variables that are not influenced by human pressures avoids this issue. Thirdly, the use of spatially continuous variables enables predictions to be mapped to stream networks. This can broaden the application of bioassessment data to answer important stream management questions that require spatially explicit outputs [24], such as predicting responses to future climate or restoration scenarios; mapping aquatic biodiversity and identifying species conservation priorities; and predicting the invasion potential of alien or translocated species.

### Prediction of rare species

On average, rare species were slightly, but not significantly better predicted by the single species ensemble model relative to the other models. This differs from reports that rare species are better predicted by multi-species response models [25, 55] or community models [73], which directly include co-occurrence information. However, our findings concur with other studies [17, 36] where single species models slightly better predicted rare species than their community classification and multi-species response model counterparts, respectively. Possible explanations for our findings are: (1) several rare species in our study have strict habitat requirements and were consequently predicted well by all modelling strategies (e.g. *Galaxias olidus* is restricted to high elevation, low temperature areas; *Ophisternon* sp. generally occurs in low gradient, tannin stained, coastal stream habitats); (2) species co-exclusion may be indirectly inferred via absence data for the single species models (e.g. expressed by environmental conditions that favour a competitor or predator) and (3) gains in predictive accuracy made via the ensemble process may outweigh any potential gains made by inclusion of species co-occurrence information. As suggested by Hallstan et al. [17], predictions from models that use co-occurrence information may also be improved by incorporating potentially interacting species from the broader aquatic community, such as invertebrates, macrophytes, phytoplankton and piscivorous birds.

### Fish assembly and model accuracy

The strategies that allowed modelling of species-specific responses (i.e. ensemble and multi-species response models) outperformed the community/RIVPACS method across most evaluation metrics. There are two likely explanations for this. Firstly, each species may be responding independently to environmental gradients, and the RIVPACS approach, which models the response of assemblage groups, cannot reliably quantify these relationships. Secondly, the role of contemporary species interactions may not be a large component of the realised fish distribution, at least in the context of the study area. The relative roles of abiotic versus biotic drivers of riverine fish assemblage composition have been the focus of many studies (see review by Jackson et al. [74]). Generally, the emphasis of these roles are dependent on the spatial scale of the study, with abiotic factors usually being identified as being more important than biotic factors for broad scale studies such as ours. Many streams of the present study area are hydrologically variable and unpredictable, both seasonally and inter-annually [5, 45]. Consequently, biotic interactions may be superseded by the effect of strong hydrological controls which may prevent species abundances from reaching carrying capacity [74]. Additionally, the relatively unpredictable environmental conditions in our study area may have historically extirpated species, as evidenced by relatively depauperate fish assemblages by global standards [43], leading to ‘vacant niches’, thereby reducing contemporary competitive or predation pressure.

### Model appraisal for stream bioassessment

Both RIVPACS type models had low sensitivity (thus a high omission error rate), coupled with high specificity. This increases the chance of committing a type II error, that is, designating an impaired site as being unimpacted or underestimating the magnitude of prevailing impacts [5, 75]. This trade-off between specificity and sensitivity is highlighted by examining the number of taxa never predicted to occur in the training dataset for each model using O/E_50_: 16 of the 25 modelled taxa are never predicted in either RIVPACS model, whereas the ensemble model predicts all 25 taxa to occur at one or more sites. Hypothetically, 64% of species could become locally extinct without a change in the O/E_50_ metric using the RIPVACS models. Conservative models like these are potentially useful when high confidence is needed to designate a site as impaired (i.e. high specificity; low type I error rate) such as when a site is the unit being assessed for compliance (e.g. breaching of acceptable limits–[76]). When the catchment is the unit of assessment, as is often the case for broad scale bioassessment programs, more sensitive models would be desirable.

High taxa omission error rates can reduce the potential sensitivity of the O/E index to environmental impacts because rare taxa that may respond to subtle anthropogenic impacts are often excluded [75, 77]. This effect may be particularly acute for bioindicator groups with low taxa richness [4, 5], as is the case in the present study. Typically, researchers have focussed on the trade-off between potential RIVPACS-type model sensitivity and taxa inclusion versus model accuracy and precision by testing these responses at various probability of occurrence cut-off thresholds (0.1, 0.2, etc.) (e.g. [78]). The decision of whether or not to include rare species is the subject of ongoing debate [77, 78, 79, 80]. We suggest the following three options for optimising this trade-off. Firstly, alternative modelling strategies to RIVPACS community type models should be appraised, as in the present study and several others [17, 20, 21]. Secondly, certain taxa may be omitted on the basis of taxa specific evaluation metrics (e.g. AUC, CCR) so that rare, but accurately modelled taxa can be distinguished from statistically ‘noisy’ taxa such as those that are unreliably sampled or governed by stochastic rather than strong niche related processes. In our study, the multi-species response models did this inherently; for example, the MANN model did not make predictions for four of the five least accurately predicted species across all models (using AUC). Thirdly, all taxa can be included with optimised thresholds applied on a per-taxon basis rather than applying a consistent threshold across all taxa (e.g. [81]). This approach has been adopted for converting probabilities to presence-absence data in most recent SDM studies (e.g. [31, 35, 36]), and thresholds can be optimised for a range of model evaluation metrics. Ultimately, the choice of evaluation metric depends on the intended application of the model [29, 82]. In the present study, we chose a cut-off threshold of 0.5, as this is the most common threshold used in RIVAPCS type models. However, this does not necessarily preserve the taxa prevalence of the training data, or result in the highest prediction accuracy, particularly for taxa with high or low prevalence [82]. Further work is required to determine if using taxon-specific thresholds, rather than an arbitrary threshold of 0.5, can improve model accuracy for bioassessment.

In general, the ensemble model provided accurate and precise estimates of O/E_50_, while affording the greatest number of species available for prediction relative to the other strategies. While relatively high precision was maintained across external datasets, mean O/E_50_ and regression slope were consistently <1 (Linke et al. [83] recommend a slope between 0.85 and 1.15). Given that mean AUC values were always highest for the ensemble method, we do not attribute this to model over-fitting; instead we suggest that the model is sensitive to assemblage changes through time (e.g. seasonal migrations of certain species such as *Percalates novemaculeata* and *Trachystoma petardi* which were both more prevalent in the post-wet samples in SEQ), and sampling differences (method dataset).

The two multi-species response models were also relatively precise and accurate for O/E_50_, but were less biased for external datasets than the ensemble model, and included more potential taxa than the two RIVPACS models. These models also performed better for the external ‘space’ dataset than the ensemble model; this is important given that accurate predictions are needed for new test sites. For these reasons, we recommend that either the MANN or MARS O/E_50_ model outputs would be most appropriate for bioassessment in our situation, if the taxonomic completeness (O/E) index was to be used.

The taxonomic completeness index disregards information about species that were observed but not predicted [17] and for this reason, differences in AUC, Kappa and sensitivity among models were not mirrored in O/E_50_ values. In contrast the BC index does rely on this information, and is the probable reason that the ensemble model produced the lowest mean BC values. BC has been proposed as complementing taxonomic completeness because (1) it includes low probability taxa and (2) it can detect stress-induced assemblage shifts that do not affect taxa richness [61]. If the BC index was to be used for bioassessment in our situation, the ensemble model would be the most appropriate choice.

In general, the models derived from a single season (winter 2013), were able to predict assemblage composition with reasonable accuracy for sites sampled in different seasons and years, and using a different sampling protocol (Table 4). This is important, as the study area contains streams with that exhibit a high degree of hydrological variability, and ideally, bioassessment is able to distinguish anthropogenic from natural stressors. A similar study in southeast QLD [6], also found that fish assemblage predictions from a community classification model were reasonably stable through time, although cautioned against relying on assessments from sites experiencing extended low flow periods (e.g. leading to isolated pools and restricted fish movement).

### Matching modelling strategy, evaluation metrics and application

By assessing several different modelling strategies using multiple evaluations metrics, stream managers can appraise the value of models for different stream management applications [16]. For example, a model with high sensitivity would be desirable for determining site susceptibility to an invasive species, because of the high error cost associated with predicting unsuitable habitats incorrectly. Conversely, if the model purpose was for designating critical habitat for a threatened fish species, the focus might be on obtaining high specificity, because of the high cost associated with protecting sections of streams where the target species does not inhabit. In our study, ensemble models had high AUC, kappa and sensitivity values across all datasets, without significant reductions in specificity relative to other models. Therefore, we suggest that the ensemble modelling strategy would be superior for most of the non-bioassessment applications (Table 7).

**Table 7:** Desirable model evaluation properties for several common stream bioassessment applications of SDMs.

Model Application	Desirable evaluation properties	Notes/references
Bioassessment (reporting at the catchment scale)	Balanced Sp and Se, mean O/E close to unity, low BC, High r^2^, low SD, low bias, low omission rate for assemblages with few taxa.	Balance between type I error (incorrectly diagnosing an impaired stream as ‘reference’) and type II error (incorrectly diagnosing a reference stream as impaired). Low availability of modelled taxa can lead to coarse estimates of ecological condition [4].
Bioassessment (regulatory/compliance, at the site scale)	High Sp, mean O/E close to unity, low BC, High r^2^, low SD, low bias.	Certainty of species loss is required to be confident that acceptable limits have been breached
Biodiversity mapping	Balanced Sp and Se, high r^2^, low SD, low bias, O/E_0_ close to unity	Requires a good regression fit of O vs. E
Species conservation and reserve design	High Sp	High commission errors may lead to protection of habitats where target species may not actually inhabit (leading to potentially wasted limited resources) (e.g. [84])
Population discovery and range extension (survey gap analysis)	High Se	Commission errors are acceptable (e.g. accurate model, incomplete data such as species difficult to sample efficiently)
Climate change	Balanced Sp and Se, or high Se	[2] notes that guidance on whether to balance errors or down-weight commission errors is unresolved.
Restocking and translocation suitability; habitat restoration	High Se	Focus on low omission error because species absences may be due to impacts and local extinctions (usually the impetus for restocking/restoration)
Predicting site susceptibility to invasive species	High Se	Omission errors are less acceptable because of the costs associated with incorrectly identifying unsuitable habitat.

Notes are sourced from [2], [16], and [83]. Refer to Table 1 for a description of the model evaluation property acronyms.

## Conclusions

We suggest that the multi-species response modelling strategy is most suited to bioassessment using freshwater fish assemblages in our study area. At the species level, the ensemble model exhibited high sensitivity without reductions in specificity, relative to the other models. We suggest that this strategy is well suited to other non-bioassessment stream management applications, e.g. identifying priority areas for species conservation. However, it must be stressed that these findings are specific to the datasets used, taxa, and study area. Further studies are required before making generalisations about which modelling strategies are most appropriate for specific stream management applications, if generalisations can be made at all. We agree with Olden and Jackson [16] in advocating that several modelling strategies should be evaluated on the same dataset so that model outputs can be matched to intended uses. We believe that by exploring alternative modelling strategies, bioassessment data can answer many important stream management questions beyond the scope of ‘first cut’ river health assessments.

## Supporting Information

S1 Fig Cluster dendrogram showing the 6 groups (red boxes) selected for the community classification models, RF and DFA. (DOCX)

S1 Table Fish presence-absence data, environmental predictor variables and site locations for the ‘training’, ‘space’, ‘season ‘, ‘time’ and ‘method’ datasets. The ‘time’ and ‘method’ datasets were made available by SEQ Healthy Waterways Partnership and NSW DPI Fisheries, respectively.
(XLSX)

S2 Table Details of predictor variables used for the fish species distribution modelling and rationale for their selection. (DOCX)
