# Supplementary figures and images for: Testing Three Species Distribution Modelling Strategies to Define Fish Assemblage Reference Conditions for Stream Bioassessment and Related Applications
Source: PLoS One. 2016 Jan 12;11(1):e0146728. doi: 10.1371/journal.pone.0146728 (PMC4710458; doi:10.1371/journal.pone.0146728)

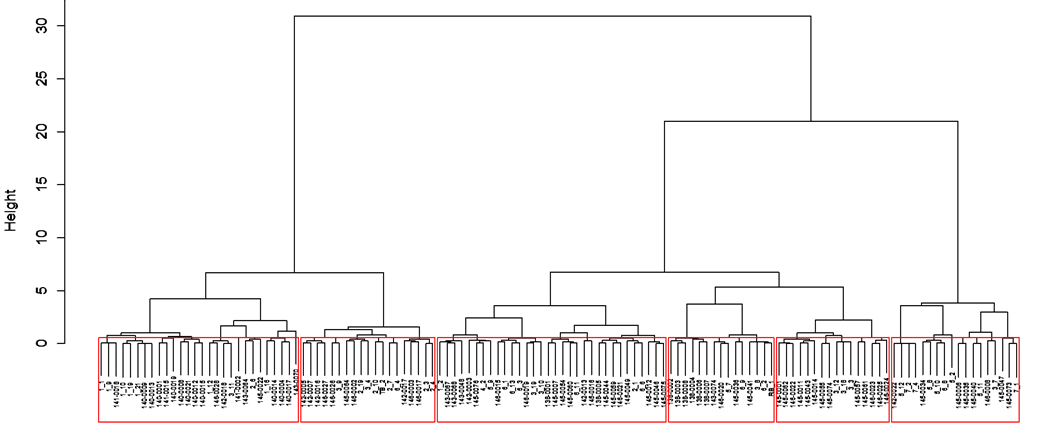
S1 Figure

Supplement: S1 Fig — (DOCX) [file pone.0146728.s001.docx]
