# Supplementary material for: Testing Three Species Distribution Modelling Strategies to Define Fish Assemblage Reference Conditions for Stream Bioassessment and Related Applications
Source: PLoS One. 2016 Jan 12;11(1):e0146728. doi: 10.1371/journal.pone.0146728 (PMC4710458; doi:10.1371/journal.pone.0146728)
Supplement: S2 Table — (DOCX) [file pone.0146728.s003.docx]

S2 Table

| **Spatial scale** | **Description** | **Units** | **Transformation** | **Rationale** |
| --- | --- | --- | --- | --- |
| Site | Mean wetted stream width | m | **-** | Indicative of position in stream continuum (Vannote *et al*. 1980) and habitat availability |
|  | Mean stream depth | cm | LOG_10_ | Proxy for habitat and cover availability, particularly for large bodied species |
|  | Mean flow velocity | ms^-1^ | LOG_10_ (x+1) | Indicative of hydraulic flow types at a site (e.g. presence of riffle/run/pool habitat) |
| Stream Segment | Mean segment elevation | m.a.s.l | - | Proxy for position in stream continuum and stream temperature |
|  | Stream and sub-catchment hottest month mean temperature | °C | - | High stream temperatures may exceed species tolerance range for survival/growth/reproduction |
|  | Stream and sub-catchment average annual rainfall | mm | LOG_10_ | Proxy for flow conditions, available aquatic habitat and surrounding vegetation types |
|  | Stream and valley percentage unconsolidated rocks | % | - | May affect water chemistry and substrate type and is related to position in stream continuum |
|  | Stream and valley percentage siliciclastic/undifferentiated sedimentary rocks | % | - | May affect water chemistry and substrate type |
|  | Stream and valley percentage metamorphic rocks | % | - | May affect water chemistry and substrate type |
|  | Stream and valley percentage mixed sedimentary and igneous rocks | % | - | May affect water chemistry and substrate type |
| Downstream flow path | Average slope of downstream flow path | % | LOG_10_ | Proxy for position in stream continuum and potential natural barriers to upstream migration |
|  | Distance to outlet (the sea) | km | LOG_e_ | Access to the sea is required for diadromous species |
| Upstream catchment | Annual mean accumulated soil water surplus | ML | LOG_10_ | Proxy for position in stream continuum, flow permanence; and available habitat |
|  | Coefficient of variation of monthly totals of accumulated soil water surplus | N/A | LOG_10_ | Flow predictability can favour species with certain life history traits |
|  | Maximum upstream elevation | m.a.s.l | - | Total available upstream habitat for colonisation potential |
|  | Catchment percentage unconsolidated rocks | % | - | May affect water chemistry, substrate type and relate to position in stream continuum |
|  | Catchment percentage igneous rocks | % | - | May affect water chemistry, substrate type and relate to position in stream continuum |
|  | Catchment percentage metamorphic rocks | % | - | May affect water chemistry, substrate type and relate to position in stream continuum |
|  | Catchment percentage mixed sedimentary and igneous rocks | % | - | May affect water chemistry, substrate type and relate to position in stream continuum |
|  | Catchment shape (elongation ratio) | N/A | - | May affect peak times of high flow events and potentially stream temperature |
|  | Catchment relief ratio | N/A | Square-root | Proxy for position in stream continuum, substrate size distribution, flow types |
|  | Catchment average slope | ° | - | Proxy for position in stream continuum and diversity of hydraulic flow types |
|  | Modelled annual terrestrial mean net primary productivity | tC ha^-1^ | - | Proxy for stream productivity and food availability |

References

Vannote RL, Minshall GW, Cummins KW, Sedell JR, Cushing CE. The river continuum concept. Canadian journal of fisheries and aquatic sciences. 1980; 37(1): 130-13
